# Supplementary material for: SARS-CoV-2 seroprevalence in a strictly-Orthodox Jewish community in the UK: A retrospective cohort study
Source: Lancet Reg Health Eur. 2021 May 18;6:100127. doi: 10.1016/j.lanepe.2021.100127 (PMC8291041; doi:10.1016/j.lanepe.2021.100127)
Supplement: Supplementary file 1 [file mmc1.docx]

Caption for supplementary material

1. Supplementary Appendix
2. STROBE Checklist
