# Supplementary material for: SARS-CoV-2 seroprevalence in a strictly-Orthodox Jewish community in the UK: A retrospective cohort study
Source: Lancet Reg Health Eur. 2021 May 18;6:100127. doi: 10.1016/j.lanepe.2021.100127 (PMC8291041; doi:10.1016/j.lanepe.2021.100127)
Supplement: Supplementary file 2 [file mmc2.docx]

**Supplementary Information for**

**Extremely high SARS-CoV-2 seroprevalence in a strictly-Orthodox Jewish community in the UK**

**Authors:** Katherine M Gaskell^1^, Marina Johnson^2^, Victoria Gould^1^, Adam Hunt^2^, Neil RH Stone^1,3^, William Waites^4^, Ben Kasstan^5^, Tracey Chantler^6^, Sham Lal^1^, Chrissy h. Roberts^1^, David Goldblatt^2^, Rosalind M Eggo^4^ and Michael Marks^1,3^

1. Department of Clinical Research, London School of Hygiene & Tropical Medicine, Keppel Street, London. WC1E 7HT UK
2. Great Ormond Street Institute of Child Health Biomedical Research Centre, University College London
3. Hospital for Tropical Diseases, University College London Hospital NHS Foundation Trust, London
4. Centre for Mathematical Modelling of Infectious Diseases, London School of Hygiene & Tropical Medicine, Keppel Street, London. WC1E 7HT UK
5. Centre for Health, Law and Society, University of Bristol Law School, Bristol. BS1 1RJ
6. Department of Global Health and Development, London School of Hygiene & Tropical Medicine, Keppel Street, London. WC1E 7HT UK
7. **Demographics**

**Supplementary Table 1: Survey respondent demographics**

| **Variable** | | **Frequency** |
| --- | --- | --- |
| **Sex** | Male | 853 (48.5%) |
|  | Female | 906 (51.5%) |
| **Age** | Median (IQR) | 14 years (7-33) |
| **Age Group** | Early Years (0-4 years) | 307 (17.5%) |
|  | Primary School (5-10 years) | 357 (20.3%) |
|  | Secondary School (11-18 years) | 360 (20.5%) |
|  | Adults (19-66 years) | 684 (38.9%) |
|  | Retirement Age Adults (67+) | 51 (2.9%) |
| **Education and Employment** | In formal education | 776 (44.1%) |
|  | Working from home | 238 (13.5%) |
|  | Working outside home | 133 (7.6%) |
|  | Neither in education or formal employment | 612 (34.8%) |
| **Self Reported Comorbidities** | Asthma | 11 (0.6%) |
|  | COPD | 2 (0.1%) |
|  | Hypertension | 31 (1.8%) |
|  | Diabetes | 21 (1.2%) |
|  | Cardiovascular Disease | 9 (0.5%) |
|  | Chronic Kidney Disease | 1 (0.1%) |
|  | Dementia | 0 (0%) |

1. **Antibody Titres and time since self-reported COVID-19-like symptoms**


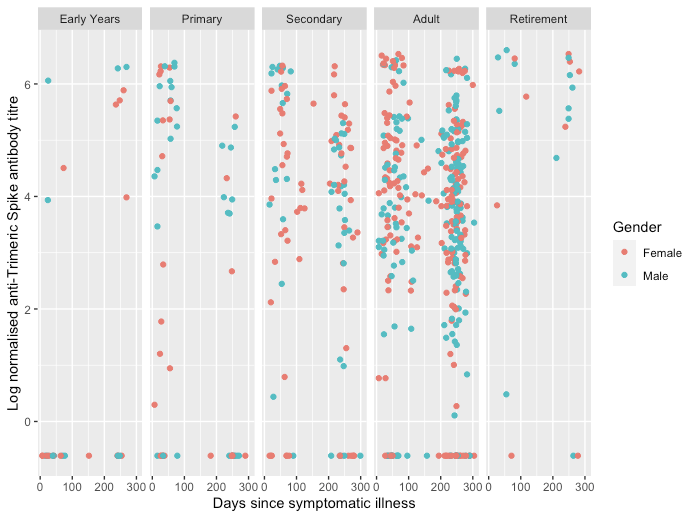


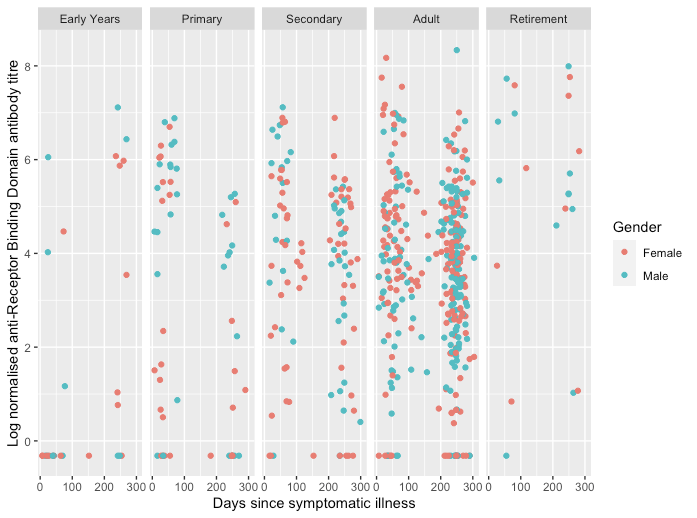


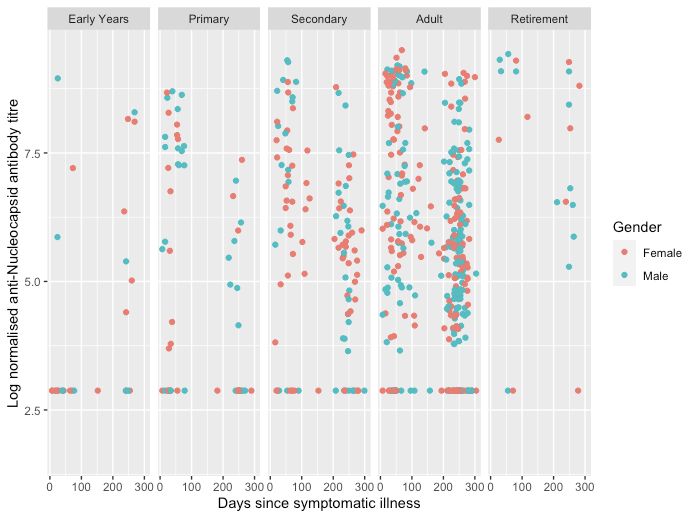


**Supplementary Figure 1. Log normalised antibody titres against spike, receptor binding domain and nucleocapsid antigens by time since self-reported COVID-19 symptoms.** Values are shown stratified by age (panels) and sex (colour).

1. **Antibody Seroprevalence by antibody target and age group**

**Supplementary Table 2: Age stratified seroprevalence.**

| **Age Group** | **anti-Spike SARS-CoV-2 antibodies** | **anti-Receptor Binding Domain antibodies** | **anti-Nucleocapsid SARS-CoV-2 antibodies** |
| --- | --- | --- | --- |
| Early Years (0-4 years) | 27.6% (20.8-35.6%) | 22.4% (16.2 - 30.0%) | 18.4% (12.8 - 25.7%) |
| Primary School (5-10 years) | 56.4% (49.8-62.7%) | 43.8% (41.8 - 54.9%) | 42.8% (36.4-49.4%) |
| Secondary School (11-18 years) | 73.8% (68.2-78.8%) | 65.6% (59.7 - 71.1%) | 50.9% (44.9-56.9%) |
| Adults (19-66 years) | 74% (70.0-77.6%) | 57.8% (53.5-62.0%) | 45.4% (41.1-49.7%) |
| Retirement Age Adults (67+) | 54.8 (38.8-69.8%) | 40.5% (26.0-56.7%) | 45.2% (30.2-61.2%) |

1. **Positive and negative predictive value of symptoms by age group**

**Supplementary Table 3: Positive and negative predictive values stratified by age for symptoms reported as COVID-19-like illness.** PPV = positive predictive value, NPV = negative predictive value.

|  | **Fever** | | **Cough** | | **Loss of Smell or Taste** | |
| --- | --- | --- | --- | --- | --- | --- |
| **Age Group** | **PPV** | **NPV** | **PPV** | **NPV** | **PPV** | **NPV** |
| Overall | 79.0% | 38.3% | 81.6% | 43.6% | 94.1% | 42.7% |
| Early Years (0-4 years) | 33.3% | 68.2% | 36.4% | 76.1% | 33.3% | 68.1% |
| Primary School (5-10 years) | 57.1% | 42.9% | 65.2% | 48.3% | 100% | 44.6% |
| Secondary School (11-18 years) | 63.0% | 25.0% | 73.0% | 28.0% | 88.9% | 29.1% |
| Adults (19-66 years) | 90.9% | 31.0% | 91.4% | 35.9% | 96.6% | 37.1% |
| Retirement Age Adults (67+) | 77.8% | 51.5% | 90.0% | 56.3% | 80.0% | 48.6% |

1. **Sensitivity Analysis**

In a sensitivity analysis in which the threshold for spike positivity was doubled, seroprevalence was 49.7%.

1. **Population Structure**

**
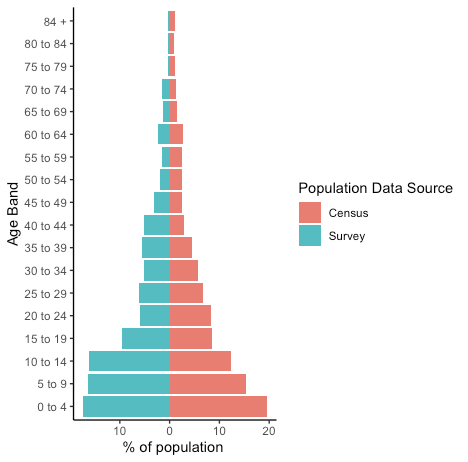
**

**Supplementary Figure 2. Population age structure for survey respondents compared to the overall Haredi population.** Census data is from 2011.

1. **Consort Diagram**


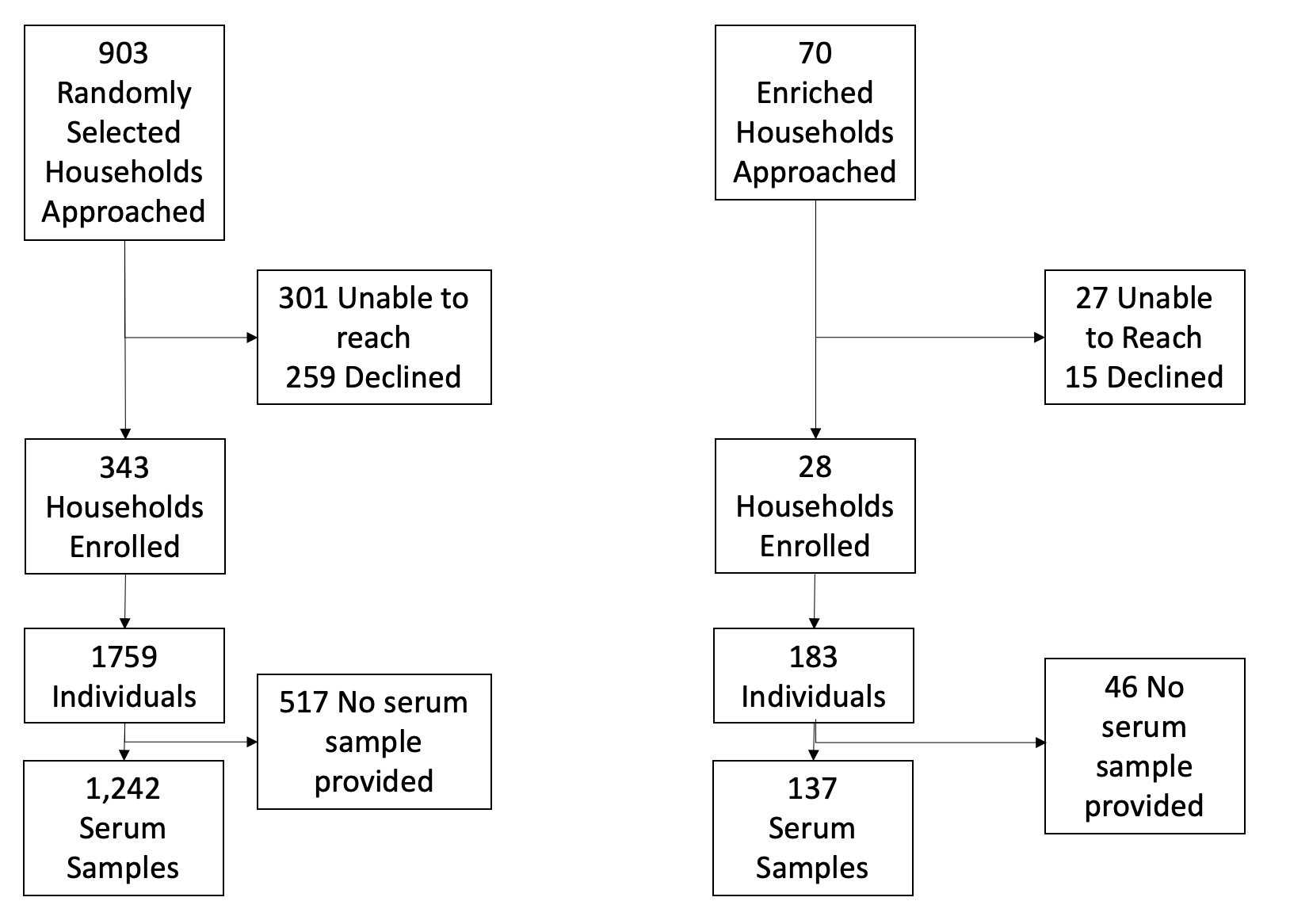


**Supplementary Figure 3. Consort diagram showing enrollment of both the randomly selected and the enriched households into the study**
